# Laboratory Diagnostics of Primary Hyperaldosteronism and its Peculiarities (Literature Review)

**DOI:** 10.25122/jml-2019-0073

**Published:** 2019

**Authors:** Viktor O Shidlovskyi, Oleander V Shidlovskyi, Mikhail Sheremet, Igor V Zhulkevych, Sergyi M Andreychyn, Inna I Hanberher, Ivan I Smachylo, Volodimir B Dobrorodny, Yuryi M Futuima

**Affiliations:** 1. 1st Surgery Department of I. Horbachevsky Ternopil National Medical University, Ternopil, Ukraine; 2. 1st Surgery Department of Bukovinian State Medical University, Chernivtsi, Ukraine

**Keywords:** primary hyperaldosteronism, nosological forms, topical diagnosis

## Abstract

The final stage of the diagnostic of primary hyperaldosteronism is to identify the causes of excessive secretion of aldosterone and determination of its variants. Based on the analysis of literature data, the diagnostic value, sensitivity and specificity of the methods of radiation diagnostics for primary hyperaldosteronism were assessed: ultrasound, computed tomography, magnetic resonance imaging, photon emission tomography, magnetic resonance spectroscopy, scintigraphy with iodine radiopharmaceuticals. The causes of false-positive and false-negative evaluations of changes in adrenal glands in the application of these diagnostics have been analyzed. There are many genetic and morphological studies when searching the literature data on the principles and methods of distinguishing the nosological forms of primary hyperaldosteronism based on the results of the aldosterone level estimation in the separated blood from the central veins of both adrenal glands or segmental veins of one gland with subsequent determination of the concentration gradient. It was noted that topical diagnostics and, especially, the determination of nosological forms of primary hyperaldosteronism are complex and expensive, but their results allow choosing an appropriate treatment approach for each particular case.

## Introduction

The main task of topical diagnosis is the visualization of the pathology of adrenal glands. It is performed using radial detection methods such as ultrasound, computed tomography, magnetic resonance imaging, positron emission tomography, scintigraphy with iodine-based radiopharmaceuticals.

Ultrasound investigation is a widely recognized diagnostic tool in today’s clinical practice and the first method of visualization when searching for diagnosis [1, 2]. The absence of factors such as radiation load, the use of contrast agents, the independence of the informativeness of the study from the functional state of the investigated organs, the possibility of multiple use of the method and the economic availability make ultrasound scan the “first echelon” in the examination of patients with adrenal tumors. This routine diagnostic method has become today the “gold standard” for the study of all parenchymal organs of the abdominal cavity and retroperitoneum, including the adrenal glands.

In many publications, the results of ultrasound scans for the detection of focal pathology of the adrenal glands are estimated to be reliably high, and in some cases, its sensitivity and specificity reach 100 % [3]. It allows estimating the size of tumors greater than 1 cm. The sensitivity and specificity of the method in the differential diagnosis of morphological types of hyperaldosteronism and other benign hormonally active tumors are not high and, according to many publications, they are estimated to be about 70%. These hormonally active tumors, such as aldosteronoma, pheochromocytoma and corticosteroma, are characterized by reduced echogenicity, have a regular round or oval shape, with clear, even contours [5]. In the case of adrenocortical cancer of small size (1-2 cm), the echographic picture does not differ significantly from that of tumors of other origins [6]. False-negative results of ultrasound examination of the adrenal glands are associated with many factors. Among them there is a close adherence of the right adrenal gland to the posterior lower surface of the right lobe of the liver, the placement of the lower part of the anterior surface of the left adrenal gland above the upper surface of the pancreatic tail adherence of the lower surface of both adrenal glands to the upper poles of the kidneys, and in case of lower positioning – partial or full adherence to the elements of the renal hilum, especially to the left. Thus, despite the accessibility and ease of examination, ultrasound is not the most informative method of diagnosis of adrenal pathology. Furthermore, it is considered a screening method in the diagnosis of aldosteronoma and its morphological subtypes because of its low sensitivity and specificity [7].

Computed tomography in the topical diagnosis of all types of adrenal cortex tumors was recognized as one of the most modern methods. It allows determining the size of the tumor, its density, structure, relationship with neighboring organs, and blood vessels. The addition of its intravascular enhancement makes it possible to detect hyperplasia or to determine the localization and characteristics of the tumor. In this case, the sensitivity of computed tomography is about 84.3 % [8, 9].

Compulsory conditions for computed-tomographic scanning are slice thickness of 2-2.5 mm and the use of vascular enhancement. Depending on the results, one can find a normal adrenal structure, unilateral macroadenoma (more than 1 cm), unilateral microadenoma (less than 1 cm), minimal unilateral or bilateral thickening of the adrenal glands, bilateral macro- or microadenomas or various combinations. Aldosteronomas are usually small in size, unlike other adrenocortical adenomas.

• According to the results of the computed tomography scan, false-positive or false-negative evaluations of changes in the adrenal tissues, especially hyperplasia, which may accompany adenoma and may be considered as idiopathic bilateral hyperplasia, are possible. Similarly, since prolonged hyperplasia of the adrenal glands is associated with the formation of pseudo-nodal lesions, this radiographic picture is often considered to be an aldosterone-producing adenoma [10, 11]. Additionally, it is necessary to keep in mind the cases of unilateral macroadenoma that is nonfunctional, and that hyperaldosteronism may be responsible for minor hormonal hyperplasia of the same or opposite adrenal gland.
• Adrenocortical cancer with hyperproduction of aldosterone is usually evaluated as a tumor with a diameter greater than 4 cm. In this case, it is possible to detect signs of suspicion about the malignant nature of the tumor (high density, delayed contrast washout) [12]. Most of the benign, unilateral adenomas secrete aldosterone or cortisol.
• Small aldosteronomas can be considered as hyperplasia, especially in the bilateral process or multiple nodal lesions, which are not identified due to the small size. In addition, the so-called ‘obvious adenoma of adrenal glands’ may be sites of focal hyperplasia. Unilateral hormonally inactive adrenal macroadenomas are relatively typical for patients over 40, and they are no different from aldosterone-producing adenomas in computed tomography. Meanwhile, unilateral hyperplasia may show an increase in the size of the gland, or it is visualized as unchanged [13].
• In the literature, based on the analysis of data on the use of computed tomography in the diagnosis and differential diagnosis of tumors of adrenal glands, the following tactics are defined:

1. In the case when the formation of the adrenal gland is diagnosed earlier by other methods of research, computed tomography is recommended with low slice thickness and bolus tracking. An asymptomatic lesion less than 3 cm in size, with a computed tomography density lower than 10 HU, can most likely be considered benign. CT washout equal to or greater than 60% confirms that the lesion is benign.

2. In the case of asymptomatic lesions that are detected by chance during a computed tomographic examination of the abdominal cavity organs and retroperitoneal space with contrast enhancement, a delayed scan at 15 minutes is performed. If a relative enhancement washout of 40% or higher is achieved after 15 minutes, the lesion is considered benign. Absolute values of density during the delayed phase were unreliable in the differential diagnosis.

3. Heterogeneous lesions of more than 3 cm in size, detected during a computed tomography scan, which accumulate contrast agent, may cause suspicion of malignancy.

4. In patients with an oncological medical history, if focal lesions of the adrenal glands are detected, one should take into account their possible metastatic nature. Computed-tomographic signs of such lesions will be variable, depending on the computed tomography pattern of the primary tumor.

5. If the computed-tomographic sign of the lesion does not meet the criteria listed above, it should be regarded as uncertain. There are no specific recommendations for such lesions, and further tactics depend on the whole set of clinical symptoms and laboratory tests [14, 15].

## Discussion

Magnetic resonance imaging (MRI) is successfully used in the diagnosis of adrenal tumors. In general, it does not have advantages over computed tomography scans [14–16].

The main features and advantages of magnetic resonance imaging before other imaging methods include non-invasiveness, harmlessness, and the three-dimensional nature of image acquisition [7]. The sensitivity of the method is approaching 97%, but the specificity in some cases is less effective than computed tomography – just over 90%. This is a small prognostic sign for determining the extent malignant potential of defeat. Compared with computed tomography, MRI has no advantage in evaluating the forms of primary hyperaldosteronism and is characterized by a lower spatial resolution.

During dynamic MRI of the adrenal glands, as in computed tomography scans, the pronounced accumulation of the contrast material and its slow elimination in malignant formations are observed, whereas the contrast material is rapidly eliminated in benign tumors [14].

Magnetic resonance spectroscopy, developed recently, is one of the few methods that allows analyzing in vivo the chemical structure of the tissues of a living organism. Today it is the method of choice in the study of internal organs, including the adrenal glands. The method has a sensitivity of 91% and a specificity of 94% in determining the nature of the adrenal lesion in comparison with histopathological studies [17]. Analysis of literature data on the use of magnetic resonance imaging in the diagnosis and differential diagnosis of adrenal tumors allowed specialists to determine the following tactics of magnetic resonance imaging to diagnose adrenal glands tumors:

1. If the lesion of the adrenal gland has typical magnetic resonance signs of fluid without a soft tissue component, further diagnostic studies are not appropriate.

2. In asymptomatic tissue lesions having magnetic resonance signals and a structure similar to the adrenal gland tissue, a scan is performed 15 minutes after the injection of the contrast agent. The lesion is considered benign if it is characterized by relatively high washout rates of the contrast material (at 15 minutes).

3. When the lesion of the adrenal gland was pre-visualized by other methods, a scan with a minimum thickness in different planes is recommended for differential diagnosis. In the presence of a heterogeneous magnetic resonance structure of the formation, studies using contrast enhancement are conducted. If there is a pronounced washout of the contrast agent, then we can assume that the tumor is benign.

4. A large (more than 5 cm) heterogeneous lesion, which accumulates the contrast agent well and for a long time, is suspected of malignancy and requires morphological diagnosis, in particular, a fine needle aspiration biopsy.

5. If the nature of the lesion is questionable, after 3-4 months of dynamic observation, it is necessary to repeat and expand the diagnostic search [14, 18].

Thus, ultrasound examination and computed tomography play a leading role in the topical diagnosis of adrenal tumors. In the case of a large-sized tumor, angiography is the most informative test which allows determining the correct topical diagnosis in 92.6 % of cases. As an additional refining method in challenging cases, magnetic resonance imaging can be used.

Positron emission tomography is a relatively new method of instrumental diagnostics, which is based on the fact that some radiopharmaceuticals at elevated adrenal function are more likely to exhibit enhanced cellular activity than standard imaging techniques. These drugs can be directed to individual enzymes that are formed in the cells of the adrenal tumors. For example, the use of 18F-dopamine allows capturing high-resolution images and detect malignant tumors.

According to N. Ghanem et al., the use of this method allows differentiating benign from malignant lesions with high sensitivity and specificity – up to 100% [19]. The literature does not contain data on the use of positron emission tomography in the diagnosis of aldosteronoma. The method is especially useful for differential diagnosis of benign and malignant tumors in the adrenal glands, especially in patients with a medical history of cancer, or when, according to a computed or MRI scan, it is impossible to eliminate the suspicion of a malignant process.

Scintigraphy with an iodine-containing radiopharmaceutical makes it possible to determine the localization of various tumors of the adrenal glands with a diameter of 0.5 cm, as well as to detect diffuse or diffuse nodular hyperplasia of both glands in different types and subtypes of hyperaldosteronism. The essence of the method is that for the study, one can use a radioactively noticed predecessor of hormones of the adrenal cortex hormones – cholesterol. For this purpose, 131J-methylnorcholesterol (NP-59) was selected [14, 20].

On the basis of the analysis of data on the inclusion of cholesterol in the synthesis of hormones, one can estimate the localization and functional characteristics of tumors in the cortical layer. Asymmetric accumulation of the radionuclide in the tissue of both glands allows identification of aldosterone-producing adenoma. In bilateral hyperplasia of the adrenal glands, the moderate absorption of radiopharmaceuticals by both glands is indicated in 72–120 hours.

According to Di Martino et al., the sensitivity and positive predictive value of radioisotope scintigraphy were 90.9 and 83.3%, respectively. The evaluation of the efficacy, based on postoperative blood pressure monitoring, showed that the sensitivity and positive predictive value of the method were 91.6%, respectively. However, its specificity, including the diagnosis of unilateral small- and large-focal nodular hyperplasia, is approaching 100 % [21].

As a variant of scintigraphy, before its application, inhibition of the secretory function of the adrenal cortex by dexamethasone (dexamethasone suppression test) is used. In this case, the image of the adenoma remains visible due to its increased secretory activity, and the image of hyperplastic tissue disappears. It is believed that in some cases, the use of the NP-59 test for the diagnosis of aldosterone-producing adenoma may replace the adrenal venous sampling test [21].

Establishing the nosological form of hyperaldosteronism and distinguishing its subtypes is a critical step in the diagnosis.

Traditionally, primary hyperaldosteronism is classified into four main subtypes, three of which are based on pathology (aldosterone-producing adenoma, bilateral hyperplasia of the adrenal glands and adrenocortical cancer), and the fourth is genetically predetermined. There are four genetic subtypes of familial hyperaldosteronism. With the deepening of knowledge about hyperaldosteronism, it became apparent that such a division is a gross simplification, as confirmed by morphological studies, which indicate a large variety of morphological characteristics of primary hyperaldosteronism.

The aldosterone-producing adenoma is usually unilateral, but it may be bilateral, and its cellular composition varies from the cells of zona fasiculata to the cells of zona glomerulosa, the so-called ‘hybrid cells’. In turn, as a rule, hyperplasia is bilateral, but it may be either unilateral, diffuse, micronodular or macronodular. Also, it was established that aldosterone-producing adenoma, except solitary lesions, may be associated with diffuse or nodular hyperplasia within the residual ipsilateral cortex [22]. From the mentioned data, it becomes clear that the dominant or even isolated node within the removed adrenal gland may not be the only source of excessive synthesis of aldosterone.

Consequently, given these data, from a practical and clinical point of view, the differentiation of subtypes has the purpose of not trying to determine the morphological type, but the separation of bilateral, benign lesions with excessive unilateral production of aldosterone from malignant ones. Thus, it is necessary to find out which of the four subtypes of primary hyperaldosteronism is present in a single patient. The tasks of this stage are performed with the help of morphological diagnosis, genetic tests, adrenal venous sampling with measurement of aldosterone and cortisol levels.

Determination of subtypes or differential diagnosis of subtypes of primary hyperaldosteronism begins with the exclusion of its rare form caused by aldosterone-producing carcinoma. Among the methods of morphological diagnosis, the cytological examination of punctate calcifications from the adrenal glands is most commonly used. Biopsy by needle puncture is ultrasound- or computed tomography-controlled. The results are used for differential diagnostics between nonspecific lesions of the adrenal glands (metastases, infectious lesions) and tumors of the adrenal tissues [23]. Cytological diagnosis can hardly differentiate between benign and malignant lesions of the adrenal glands. However, according to Saeger et al., the use of immunocytochemical analysis can improve the results of the study [24]. Biopsy by needle puncture is justified in patients with proven malignant diseases, in which the adrenal glands are the expected site of metastasis, and if detection of metastases in the adrenal glands may affect the choice of treatment and prognosis of the disease. It is believed that when confirming the hormonal activity of the adrenal glands during the laboratory examination biopsy by needle puncture is not indicated.

Genetic testing is a component of differential diagnosis between the primary hyperaldosteronism subtypes. Familial forms of primary hyperaldosteronism are investigated using the polymerase chain reaction or other methods. It is recommended to investigate the mutation in the KCNJ5 gene in order to exclude or confirm the type III subtype [25] in children with primary hyperaldosteronism. Detection of patients with familial hyperaldosteronism type I indicates the need for family screening. However, given that this subtype of primary hyperaldosteronism is rare (<1% of all patients with primary hyperaldosteronism), the feasibility of such testing is questionable. Therefore, it is not recommended to test for the confirmation of primary hyperaldosteronism in patients younger than 20 years of age; also, only those patients with a family history of primary hyperaldosteronism or stroke at a young age (under 40 years) should be tested [26, 27].

A dexamethasone suppression test is used when suspecting familial hyperaldosteronism type 1. In this case, despite the small doses (1-2 mg per day) of dexamethasone, there is a temporary decrease in the level of aldosterone in the plasma and urine, lowering the blood pressure [28].

Adrenal venous sampling with measurement of aldosterone and cortisol levels for distinguishing unilateral from bilateral primary hyperaldosteronism is a mandatory recommendation according to the current diagnostic guidelines [26]. This method allows obtaining information regarding the functional characteristics of the adrenal glands and their lesions, to detect unilateral or bilateral excessive production of aldosterone and, in combination with imaging methods, to establish its etiology. Establishing the level of aldosterone in the blood samples selectively collected from the adrenal veins is the “gold standard” when clarifying the unilateral and bilateral nature of aldosterone secretion in patients with primary hyperaldosteronism [29].

However, in the case of patients under the age of 35 with a detected unilateral adenoma of more than 10 mm and a structurally unaltered opposite adrenal gland, adrenal venous sampling is not mandatory if the aldosterone-to-renin ratio is higher than 30 ng/dl (831 pmol/l) and hypokalemia is evident [30].

adrenal venous sampling. In some centers, it is performed in all patients who had been diagnosed with primary hyperaldosteronism [31, 32]. Some are willing to perform adrenal venous sampling randomly, especially in those cases where the results of topical diagnostic methods do not indicate the presence of the pathology, but the aldosterone and renin levels in the peripheral blood indicate primary hyperaldosteronism and also when it was caused by bilateral hyperplasia of the adrenal glands [33–35].

With regard to the differential diagnosis of primary hyperaldosteronism subtypes, it is recommended to draw blood selectively from the central and segmental adrenal veins, with the subsequent determination of the concentration gradient of aldosterone and renin plasma at different levels of the venous bed of the individual adrenal gland [36].

An essential indicator of selective blood collection is the lateralization index, which is the ratio of the concentration of aldosterone on the dominant side (with the predominant production of aldosterone) to the concentration of aldosterone on the non-dominant side [37, 38]. A lateralization index higher than two indicates the unilateral production of aldosterone, whereas a result lower than two indicates a bilateral excess secretion from the pathologically-altered adrenal glands [39, 40].

The inclusion of adrenal venous sampling data in the differential diagnosis of primary hyperaldosteronism forms effectively reduces the risk of unreasonable adrenalectomy, which is performed only on the basis of computed tomography.

The sensitivity and specificity of adrenal venous sampling for establishing the lateralization index are 95 and 100%, respectively, and 78 and 75% for computed tomography and MRI, respectively [6, 41]. It is essential to clearly understand that making only computed tomography-based diagnostics without adrenal venous sampling might be insufficient in order to determine the form of primary hyperaldosteronism; it is possible to mislead the patient about the surgical treatment and not achieve a positive therapeutic effect.

## Conclusion

It should be emphasized that the successes achieved in recent years in the topical diagnostics of adrenal tumors are due to the widespread use of ultrasound, computed and magnetic resonance imaging. At the same time, it should be mentioned that topical diagnostics and determination of the variant of primary hyperaldosteronism are sophisticated. Last but not least, their complexity lies in many morphological and genetic subtypes of the pathology, peculiarities of the morphological structure of aldosterone-producing adenoma, in particular, the abnormal presence of cells of zona fasiculata and glomerulosa. However, the accuracy of the diagnosis has a leading role in choosing the method of treatment (surgical or medication) and in its long-term results

## Conflict of Interest

The authors confirm that there are no conflicts of interest.
